# Biological normalcy

**DOI:** 10.1093/emph/eoz035

**Published:** 2019-12-03

**Authors:** Andrea S Wiley, Jennifer M Cullin

**Affiliations:** Department of Anthropology, Indiana University, Bloomington, IN 47405, USA

## DEFINITION AND BACKGROUND

**Figure 1.**
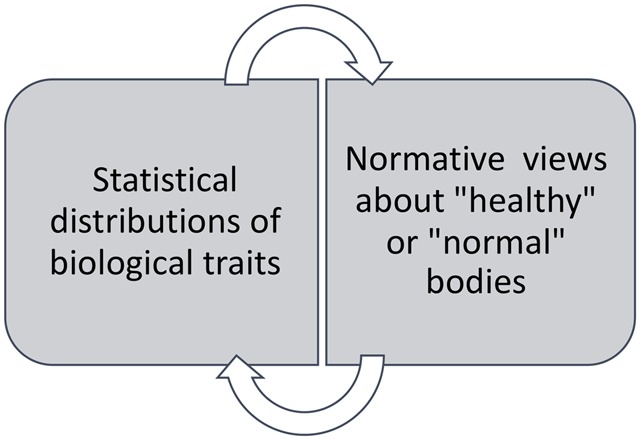
Potential relationships between statistical norms and normative views

‘Biological normalcy’ refers to relationships between statistical distributions of biological traits (measures of central tendency and variance) and normative views about what bodies ‘should’ be like or what constitutes a ‘normal’ body [1] (Fig. 1).

Statistical norms carry no explicit evaluative weight, but they may inform judgements about what is ‘normal’ or ‘abnormal’. When clinicans come from populations in which a trait is common, this may shape their cultural models of ‘normal’ human biology, especially if they lack knowledge or experience of the range of human variation.

‘Ethno-biocentrism’, which refers to cultural biases against different forms of human biology [2], can lead to pathologizing trait variants that may be adaptive. Such biases may contribute to changes in the trait’s frequency in a population due to discrimination and subsequent poor health outcomes.

Patterned human biological variation may stem from populations experiencing different selective forces over their unique evolutionary histories or current exposures to different socio-ecological conditions; thus traits should be considered within their ancestral or current environmental context.

## EXAMPLES IN CLINICAL MEDICINE AND PUBLIC HEALTH

‘Clinical trials’ historically used White adult males as ‘normal’ subjects, and only recently NIH has mandated greater inclusion of age and ethnic diversity in addition to sex/gender to gain a broader sense of ‘normal’ across the range of humanity [3]. For example, lack of research on heart disease among women leads to underdiagnosis and poorer outcomes [4].

‘Lactose intolerance’: Most humans experience a decline in the enzyme lactase in childhood. In some populations (Europeans and pastoralist groups in Asia or Africa), lactase production continues and individuals can digest the milk sugar lactose throughout their life (‘lactase persistence’). Those who are lactase non-persistent (approximately 60% of humans) are labeled with ‘lactase deficiency’, ‘lactose malabsorption’ or ‘adult hypolactasia’ in the biomedical literature, and low milk intake is blamed for health disparities [1].

‘Child growth standards’: US infants were used to establish a standard for appropriate growth, despite most being formula fed. The World Health Organization standards based on the WHO Multicentre Growth Reference Study [5] show lower rates of weight gain among healthy breastfed infants from six countries. Lower weights among breastfed infants might have contributed to increased formula usage to bring growth into line with the US standard.

‘Body weight’: Normative views about body weight are evident in public health as the BMI category deemed healthy is labeled ‘normal’, broadly pathologizing other categories. As higher BMIs become more common in many populations, high BMI individuals may come to view their own weight as ‘about normal’ [6], even while social stigma against it increases [7]. Medical professionals’ anti-fat attitudes can also result in poorer quality of care and stigmatization of patients, and perceived stigma is thought to contribute to weight gain and poorer health outcomes due to psychosocial stress.
